# Personalised Ecology

**DOI:** 10.1016/j.tree.2018.09.012

**Published:** 2018-12

**Authors:** Kevin J. Gaston, Masashi Soga, James P. Duffy, Joanne K. Garrett, Sian Gaston, Daniel T.C. Cox

**Affiliations:** 1 Environment and Sustainability Institute, University of Exeter, Penryn, Cornwall, TR10 9FE, UK; 2 Wissenschaftskolleg zu Berlin, Institute for Advanced Study, Wallotstrasse 19, 14193, Berlin, Germany; 3 School of Agricultural and Life Sciences, The University of Tokyo, 1-1-1, Yayoi, Bunkyo, Tokyo, 113-8656, Japan; 4 European Centre for Environment and Human Health, University of Exeter Medical School, Truro, Cornwall, TR1 3HD, UK

**Keywords:** ecosystem services, extinction of experience, human–nature interactions, nature–health interactions, observer bias, urbanisation

## Abstract

The field of ecology has focused on understanding characteristics of natural systems in a manner as free as possible from biases of human observers. However, demand is growing for knowledge of human–nature interactions at the level of individual people. This is particularly driven by concerns around human health consequences due to changes in positive and negative interactions. This requires attention to the biased ways in which people encounter and experience other organisms. Here we define such a ‘personalised ecology’, and discuss its connections to other aspects of the field. We propose a framework of focal research topics, shaped by whether the unit of analysis is a single person, a single population, or multiple populations, and whether a human or nature perspective is foremost.

## Highlights

Traditionally, ecologists have focused on understanding the ecological world unbiased by how human observers interact with it.

However, both the positive and negative nature interactions that people experience are the result of these biases.

The nature interactions people experience will be heavily dependent on the ecology of species, as well as their own opportunities and behaviour.

Scientists and policymakers need to determine and account for this ‘personalised ecology’, to better understand and balance the benefits that people gain from the natural world, whilst limiting negative impacts upon it.

## Human–Nature Interactions

Ecology has been defined as the study of the abundance and distribution of organisms and the interactions that determine these [1]. As such, it has been important to measure what those abundances and distributions actually are, or at least to have well behaved and characterised proxies, and to limit the influence of the human observer on these estimations. A vast and rich literature has developed particularly around the forms of biases in the human detection of individual organisms, the factors that influence those biases (individual and species characteristics, species richness, habitat, season, weather, observer skills, etc.), and the strengths and weaknesses of approaches to their reduction (e.g., 2, 3, 4, 5). Indeed, a major theme of the history of ecology as a discipline has been progressive improvement in documenting the real abundances and distributions of organisms and their respective dynamics.

By contrast, there has been little consideration of the converse need to understand the interactions that occur between human observers and nature. Nonetheless, a demand has arisen from several quarters to focus on the very effects that traditionally ecologists have sought to minimise or control in their studies. First, and perhaps foremost, it has become apparent that people derive a wide array of health and well-being benefits from their personal interactions with nature (reviewed in [6]). This is particularly so in urban areas, which are epicentres for chronic and noncommunicable physical and mental health conditions [7] and where opportunities for nature experiences may be less prevalent. These health and well-being benefits include components of mental, physical, and social health 6, 8, 9. Key to determining how these benefits are achieved is a better understanding of the form, frequency, and duration of people’s interactions with nature [10].

Second, there is growing evidence of a progressive reduction in positive human–nature interactions, particularly in more westernised societies, especially during childhood [11]. This so-called ‘**extinction of experience**’ (see Glossary) [12] results from a combination of local and regional losses of biodiversity, growth of sedentary pastimes, and perceived safety concerns that limit children’s independent activities. This may have profound consequences because the loss of human–nature interactions limits the associated health and well-being benefits. There is also evidence that it results in a reduction in emotional affinity toward nature and pro-environmental attitudes and behaviour [11]. Ongoing extinction of experience could thus imply a cycle of disaffection toward nature, and ultimately constitute one of the greatest challenges to conservation policies and management actions aimed at slowing or halting the biodiversity crisis [13]. Again, better understanding the actual nature experiences that people have and how these compare with those that are available is key to addressing these issues.

Third, there is much discussion and debate around **human–wildlife conflict**, and hence negative human–nature interactions (e.g., 14, 15). One form of this conflict concerns direct interactions between people and wildlife. In the extreme, for example, attacks on humans by large predators appear to be on the rise [16], likely as a consequence of some combination of reduction in available natural undisturbed habitat, increases in ecotourism to previously remote locations, growing familiarity of these animals with people, and inappropriate behaviour of people toward them (possibly in itself evidence of the growing extinction of experience). Other conflicts resulting from direct interactions are doubtless rife, with consequences that range from severe (e.g., emerging infectious diseases, snake bites, vector-borne disease transmission; e.g., 17, 18) to inconvenient (e.g., noise nuisance, mess and mild aggression; e.g., 19, 20). Management of these interactions are often improved by better understanding how they arise, and with what regularity.

To address this demand in a more coherent manner, we propose the need for a ‘personalised ecology’ that is distinguished by its focus on the direct interactions between individual people and nature. In this opinion article, we offer a definition of personalised ecology, suggest a framework of research topics on which personalised ecology should focus, and highlight the connections of personalised ecology to other aspects of ecology.

## Personalised Ecology

We define personalised ecology as the investigation of the direct interactions between individual people and nature and their ecological dimensions. We define nature to span individual living organisms to ecosystems, but to exclude organisms that are not self-sustained (e.g., crops, house plants, zoo and domesticated animals); we acknowledge that whilst a broadly understood distinction between these two groups is achievable, a precise and uniformly agreed one is challenging. A human–nature interaction is then a particular instance of an individual person being present in the ‘same space’ as nature or perceiving a stimulus from nature (through sight, sound, smell, taste or touch; although in practice, sight and sound tend to predominate). This might be the ecosystems that they experience, the species that they encounter, or the individual organisms they see or hear. Such an interaction could occur intentionally or unintentionally and consciously or unconsciously. To a greater or lesser extent, unconscious experiences are likely to be occurring for much of the time that people are outdoors.

A definition of this breadth allows inclusion of a wide range of types of human–nature interactions, such as visiting urban greenspaces or national parks, viewing trees through a window, listening to bird song, and being bitten by mosquitoes. It excludes interactions with nature through the media (e.g., through books, television, websites), albeit these interactions can have positive outcomes for humans (e.g., 21, 22).

The focus of personalised ecology is on the ecological dimensions of human–nature interactions, recognising that other important dimensions are not ecological and more relevant to other fields (e.g., medicine, public health, environmental education). We will also exclude for present purposes consideration of organisms that live on or in people, whilst recognising this can be a legitimate topic of ecological enquiry.

**Figure 1:**
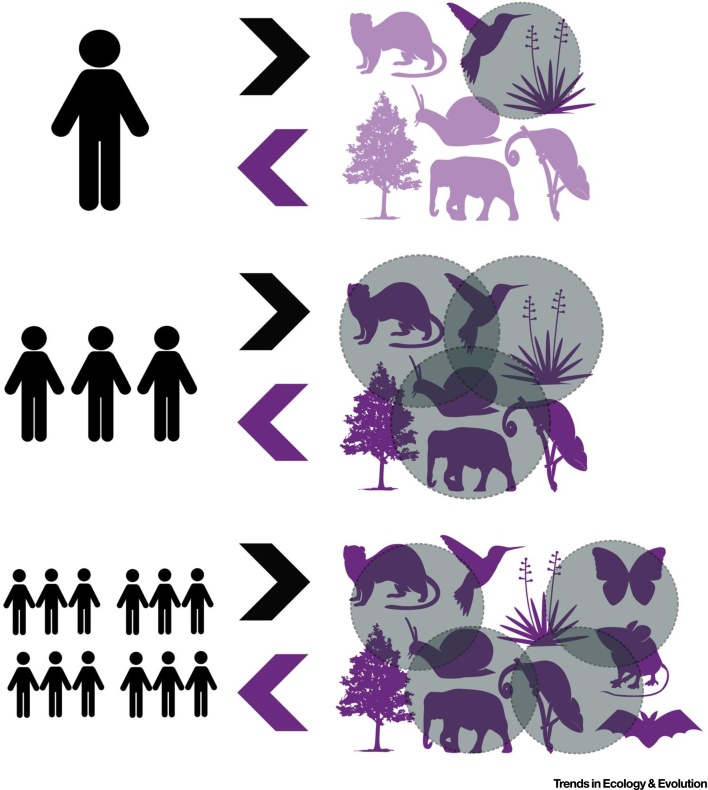
Schematic of the Different Perspectives of Personalised Ecology. Personalised ecology can be considered from the perspectives of the person, or of nature (arrows), and at different levels, namely a single person (top), a population of people (middle), or across multiple human populations (bottom). The circles represent the (overlapping) components of nature that an individual person, different people within a population, or people within different populations interact with. Note the organisms and combinations are for illustrative purposes only. Plant and animal vector images provided by http://www.vecteezy.com.

One can view personalised ecology from two perspectives; first, from that of the person, and second, from that of nature. Whilst the fundamental unit of study remains the individual person, one can consider both of these perspectives at the level of a single person, a population of people, or across multiple human populations (Figure 1). We will address each of these six combinations in turn.

### Single Person, Human Perspective

Arguably at its most reductionist, personalised ecology considers the nature that is experienced by a single individual person over a defined period. The vast majority of studies to date have simply assumed that characterisation of the environs in which people live, or of the places that they visit (e.g., public parks, protected areas), captures their experience [23]. In the main, even this has been done crudely, typically using measures of the extent of green landcover (e.g., 24, 25), although some studies have sought to characterise the abundance or diversity of taxa in these environs or places (e.g., 26, 27, 28). Undoubtedly, the actual nature interactions of people may be very different from what has typically been measured (e.g., 20, 29).

A key research focus of personalised ecology will need to be on understanding how (e.g., passively or actively) and what type of nature people are experiencing, how these experiences are influenced by personal characteristics (e.g., gender, age, observer knowledge, skills and behavioural preferences), and by the physical/environmental conditions under which these nature interactions occur (e.g., time of day, seasonality, weather). Whilst some of these factors (particularly observer skills) have been investigated in an attempt to understand the impacts on **biodiversity monitoring** schemes, the extension of these studies to a much broader cross-section of people and factors has been limited 27, 30. Nonetheless, it has, for example, been shown that ecological knowledge can be important in shaping people’s nature experiences (e.g., [31]). The continuing rapid advancement of personal monitoring devices (e.g., eye-tracking glasses, GPS trackers, electroencephalography, acoustic recorders) will enable much improved characterisation of the nature that people encounter and how this varies.

### Single Person, Nature Perspective

**Figure 2:**
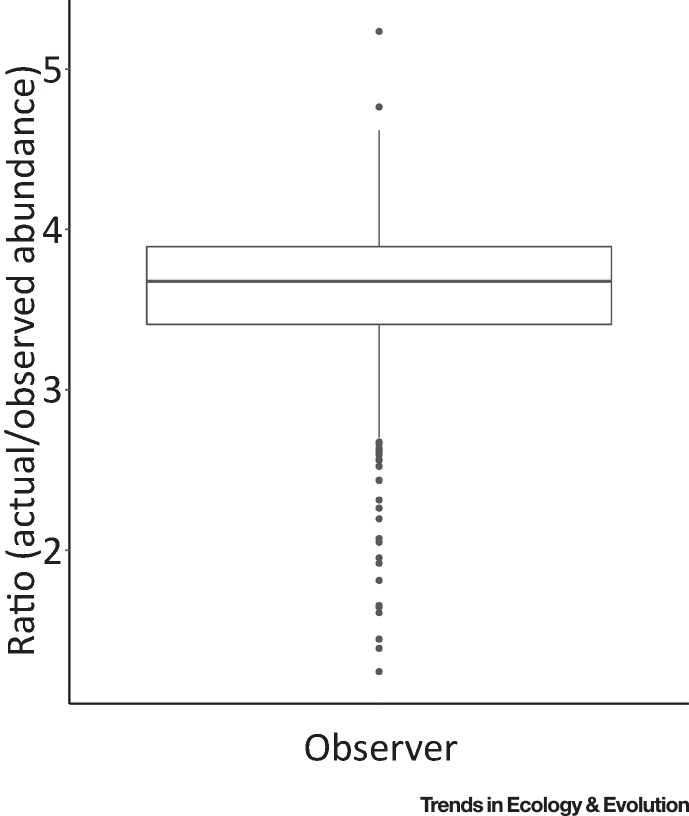
Example Variation in the Ratio of Estimated Actual Bird Abundance to Observed Bird Abundance. In total, 420 bird surveys (data from [28]) were conducted across three towns in southern England, UK. Each town was divided into 500 × 500 m tiles in a grid, with 106 tiles being surveyed. Surveys, conducted by trained observers, comprised two early-morning 10-minute point counts at up to four survey points (mean per tile, 3.91 ± 0.32 standard deviation). Actual abundances, adjusted for detection probability were then estimated from observed abundances using distance sampling (see [28] for detailed description of the methodology). The observed and adjusted abundances presented here are per survey point www.flaticon.com.

If we know which components of nature an individual person is interacting with, then we can ask how these relate to the nature that is potentially available for such experiences. The occurrence and relative frequency of interactions will almost invariably be a non-random subset of those available. For example, abundances of bird species apparent even to a trained observer will often be far less than those actually present (Figure 2). The numbers of birds that untrained people see and hear as they move around the landscape is likely to be significantly lower [32]. Such differences can arise for a diverse array of reasons, the unpicking of which may be important. These will include the actual distribution and abundance of species, their appearance and behaviour, their response to people (e.g., flight initiation distances, changes in calls), the timings of activities (e.g., daily and seasonal activity patterns, annual migration), and perceptions of where individuals are. Most obviously, people are more likely to interact with species that are common, diurnal, apparent (e.g., large, active, vocal), accustomed to people, and that can be attracted to their vicinity (e.g., through resources such as bird feeders, nest boxes).

### Single Population, Human Perspective

Within a human population, nature experiences will vary between individuals in their composition, frequency, and duration. Particularly in towns and cities, those having regular nature experiences, or ones of long duration, tend to be rare. A study in the UK found that three-quarters of direct nature interactions (instances where people were present in nature) were experienced by just one-third of an urban population [33]. As more detailed data on the nature experiences of individual people becomes easier to collect, then so too will comparisons between people. Two major sets of factors have been proposed to influence the frequency and duration of human–nature interactions. The first is the opportunity to experience nature, which is particularly shaped by the ease of access to greenspace within the local environs [34]. This can depend heavily on people’s socioeconomic circumstances. These strongly determine the kinds and location of the properties that they inhabit, and hence the availability and biodiversity of associated greenspaces 35, 36, 37, whether they can invest in green infrastructure [38] and activities to attract wildlife to those environs [39], and also whether they can engage in ecotourism elsewhere. The second influence on the frequency and duration of human–nature interactions is the orientation (or preferences) of people toward exploiting these opportunities. Although more attention has been paid to opportunity in discussions of the design of urban green infrastructure, there is evidence that orientation may be more important in shaping nature experiences [40]. These two tend to be correlated, with people living in greener areas having increased opportunity to experience everyday nature, also having a greater orientation toward doing so [41].

### Single Population, Nature Perspective

Different areas and different individual organisms will contribute very differently to the nature experiences of a given human population. Some areas will be visited by many people, others by few or none. This issue is presently best understood with regard to urban greenspaces and protected areas, where human footfall has been measured and associated with their ecological (e.g., [42]) or geographical (e.g., [40]) features. However, it remains challenging to disentangle the influence of the wide array of possible features that may determine whether areas are visited, how often, for how long, and with what consequences for nature experiences and for the management of sites (e.g., to encourage or direct access both to enhance nature experiences and mitigate impacts on wildlife). These include the sizes of areas, their accessibility, their vegetational complexity (e.g., evidence that people prefer ‘savannah-like’ natural spaces), the presence or absence of key species (e.g., large mammals), and the occurrence of wildlife spectacles. The numbers of people visiting an area will impact their individual nature experiences, due to an increase in numbers of observers (and hence what wildlife is located) and in the disturbance resulting from their activities.

**Figure 3:**
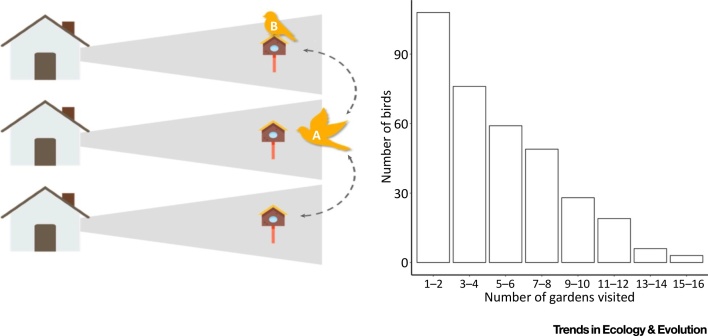
Example of Variation in the Provision of Nature Experiences Contributed by Different Individual Organisms. By moving between bird feeders in multiple gardens, bird A has the potential to be seen by more households, and thus provide nature experiences to more people than bird B, which visits only one feeder. Cox *et al.*[43] attached Radio Frequency Identification Receivers to 20 bird feeders in an equal number of gardens in three neighbourhoods in southern England (*n* = 60). They show the number of domestic gardens that songbirds carrying a Passive Integrated Transponder (*n* = 348) visited over a 12-month period. Icons provided by *Freepik* and *Smashicons* via www.flaticon.com.

Equally, there will be great variation in how species and individual organisms interact with the human population. Some individual organisms will interact with many people, others with few or none [e.g., for many years a single black-winged stilt *Himantopus himantopus*, resident on a protected area in Norfolk, UK, was held to have been watched by more people than any other bird in the country; Eele, P. (2015) Gone but never forgotten. https://ww2.rspb.org.uk/community/placestovisit/titchwellmarsh/b/titchwellmarsh-blog/archive/2015/05/21/gone-but-never-forgotten.aspx]. These experiences will be further influenced by interactions between species, which increase the probability that the organisms will encounter people or provide a more interesting spectacle. Improvements in remote sensing data and tracking technology have begun to enable evaluation of how individual organisms contribute to nature experiences [43]. In urban areas in particular, those mobile individuals that move between a greater number of greenspaces, are likely to be seen by more people (Figure 3). Similarly, individuals of those stationary organisms (e.g., trees) that are readily visible, such as beside roadsides, will be experienced by more people than others of the same or similar species.

### Multiple Populations, Human Perspective

There will inevitably be differences in nature experiences of people in different populations, such as, different villages, towns, and cities. What will be particularly important to understand is the macroecology of such variation; how the frequency, duration and composition of interactions change over large spatial and temporal scales. As with variation within single populations, opportunity and orientation will be significant, with cultural, socioeconomic, and environmental differences likely to play profound roles in shaping how people in different populations use their natural environment (e.g., [44]). However, little is known about these patterns, with the majority of studies limited to westernised countries (e.g., [45]), and so the findings may have limited generality. For example, whilst in these usually temperate zones, vegetation around the home is often seen as associated with human well-being benefits and to be encouraged (at least where it does not pose a fire risk), in many tropical areas it can harbour species dangerous to human health, and is often cleared.

Even focusing on narrow issues, approaches to nature experiences may be very different across the world. This is well illustrated with regard to attitudes toward providing supplementary food for birds and mammals in urban areas. In some parts of Europe and North America, the practice, often to increase the likelihood of viewing them, is widespread, and indeed is the basis for a substantial industry (e.g., [45]). In Australia, it is much less favoured, in part because it is seen to encourage alien or unwelcome species (e.g., [46]). In much of the rest of the world, such feeding activities are virtually unknown [47].

### Multiple Populations, Nature Perspective

When contrasting the nature experiences of multiple human populations, it seems logical to ask to what extent it is the same or analogous components of nature (e.g., the same species or species that have similar traits and ecologies) that are contributing. Such studies will be akin to those in **urban ecology** that have attempted to characterise the similarities and dissimilarities of species assemblages found in different towns and cities, albeit in this case without explicit reference to their contribution to human–nature experiences (e.g., [48]). In the main, it seems likely that species, or groups of species, that occupy similar niches in different cities will provide similar kinds of nature experiences to people. However, there are clear cases where different species fulfil the same role, with, for example, urban bird feeding tending to focus in some regions on granivorous species, and in others on nectivorous ones [47].

**Figure 4:**
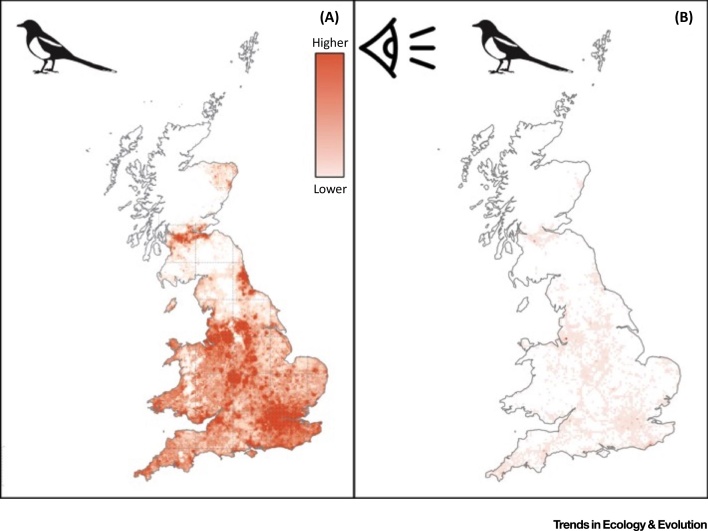
Differences between (A) the Relative Abundance of a Common, Visible, and Regionally Well-known Bird Species, the Magpie *Pica pica*, and (B) Where People Interact with this Species. (A) Shows the breeding abundance map from the Bird Atlas 2007–2011 [49], which is a joint project between the British Trust for Ornithology (BTO), Bird Watch Ireland and the Scottish Ornithologists Club (reproduced with permission from the BTO). Data were collected through ornithological volunteers carrying out bird counts in at least eight 2 km^2^ areas, within each 10 km^2^ square across the UK. (B) Shows a record of sightings collected in 2013–2014 by a much wider range of people whilst about their daily lives using the Magpie Mapper App [50]. Eye icon provided by *Freepik* via www.flaticon.com.

When looking across human populations one can start to map the spatial distribution of nature interactions, which will often be different from the underlying distributions of the species concerned. The distribution across Britain of the Magpie *Pica pica*, as recorded by citizen scientists is, for example, very different from that documented by formal ornithological mapping schemes (Figure 4). Unsurprisingly, the former highlights encounters along major transport routes and in major centres of population, as these are the places in which the vast majority of nature experiences actually occur; while the latter reveals many areas in which the species occur, but interactions are more limited.

## Linkages

Obviously, personalised ecology is not divorced from a number of other topics of focal interest in ecology. In addition to those already observed above to motivate the need for such an agenda, these include the following which are addressed in turn.

### Biodiversity Monitoring

While biodiversity monitoring has been focused principally on understanding the relationship between the actual abundances and distributions of species, and what expert observers detect, personalised ecology is less concerned with these actual quantities and more with the abundances and distributions experienced by people, and with a focus on ‘ordinary’ people (i.e., non-experts, and often with a limited knowledge of ecology), and experiences during everyday activities. The growing use of **citizen science** in biodiversity monitoring makes the concerns of personalised ecology increasingly relevant.

### Ecosystem Services

Whilst the topic of **ecosystem services** is explicitly concerned with the benefits that people gain from ecosystems [51], rather than emphasising personal nature interactions in the main this is approached in a generic sense of community or societal benefits (e.g., from agricultural production, pollination, carbon sequestration, waste decomposition). The two approaches are obviously complementary, with the ecosystem benefits to individual people often becoming very apparent in terms of cultural ecosystem services (e.g., recreational, sense of place, aesthetic, educational and therapeutic values).

### Urban Ecology

The bulk of urban ecology research remains focused on a traditional understanding of the determinants of the abundance and distribution of species, and the interactions that determine these, albeit in urban areas [52]. Nonetheless, there have been repeated calls for, and important contributions toward, broader approaches (e.g., 53, 54), and particularly those that address the complex interplay between people and urban ecosystems. Personalised ecology would clearly contribute to such an agenda.

### Human Ecology

The field of **human ecology** studies the relationship between humans and their environment, and typically has a strong emphasis on the anthropological, social, or political dimensions to this interaction [55]. Personalised ecology would again serve to add an important dimension to such investigation, by strengthening the links to more conventional ecological concerns.

## Implications

A well-developed understanding of personalised ecology would have major practical consequences in two primary arenas. First, it would improve the ability to design policy and management for people’s access to nature in such a way that their benefits (i.e., the positive interactions), were enhanced, and their costs (i.e., the negative interactions), were reduced. Second, and more importantly in the face of a global biodiversity crisis, well developed understanding of personalised ecology would improve the ability to determine policy and management of people’s interactions with nature in such a way that the benefits to nature were also increased, and the costs minimised. Of course, these two arenas interact, and what is presently lacking is a strong evidence-based approach for encouraging the positive engagement of people with nature, whilst promoting the conservation of populations and ecosystems.

## Concluding Remarks

The global human population is continuing to grow rapidly and become more urbanised, with people less likely to experience regular positive interactions with nature. At the same time, the importance of those interactions to human well-being is becoming increasingly apparent. It thus seems vitally important that ecologists develop a much more comprehensive and detailed understanding of those interactions, their composition, and temporal and spatial dynamics (see Outstanding Questions). Such a ‘personalised ecology’ constitutes a challenging agenda, and one that has thus far lagged far behind others in the field of ecology.

Outstanding Questions How can the suite of direct interactions between individual people and nature, and their strengths, most effectively be determined?

What are the differences between the direct interactions between people and nature that could occur, that do occur, and that the people concerned perceive to occur?

Which components of nature interactions provide the different kinds of benefits and costs to people, and how do these vary with cultural, socioeconomic, and environmental circumstances?

What role does personal nature experiences play in shaping people’s attitudes and behaviour toward biodiversity conservation?

## Glossary

**Biodiversity monitoring**: tracking the changes in the state of biodiversity.
**Citizen science**: scientific research conducted by those who are not professional scientists.
**Ecosystem services**: the benefits that people gain from the natural environment.
**Extinction of experience**: progressive loss of daily interactions between people and nature.
**Human ecology**: the study of the relationship between humans and their environment.
**Human–wildlife conflict**: interactions between people and wildlife that result in harm to either.
**Urban ecology**: the study of the abundance and distribution of organisms in urban environments.
